# A qualitative study of factors that managers in small companies consider important for their wellbeing

**DOI:** 10.1080/17482631.2023.2286669

**Published:** 2023-11-27

**Authors:** Elena Ahmadi, Daniel Lundqvist, Gunnar Bergström, Gloria Macassa

**Affiliations:** a Department of Occupational Health Science and Psychology, Faculty of Health and Occupational Studies, University of Gävle, Gävle, Sweden; b Department of Behavioural Sciences and Learning, Division of Education and Sociology, Linköping University, Linköping, Sweden; c Department of Public Health and Sports Science, Faculty of Occupational and Health Sciences, University of Gävle, Gävle, Sweden

**Keywords:** Managers, small businesses, qualitative content analysis, wellbeing, demands, resources

## Abstract

**Purpose:**

Given the importance of small businesses for society, and the significance of managers’ wellbeing for employee health, leadership, and business performance, more knowledge is needed on the sources of managers’ wellbeing. This study explored factors within the small business context that were perceived by managers to hinder or enable their wellbeing.

**Methods:**

Data were collected through qualitative semi-structured interviews with 20 managers from 12 small companies, and analysed with content analysis.

**Results:**

The factors that these managers in small businesses experienced as enhancing or hindering their personal wellbeing covered five categories: demands and resources in the daily managerial work, achievement of results, social factors, organizational factors, and individual factors.

**Conclusions:**

The specific context of managerial work in small companies encompasses unique factors. For instance, the small company managers’ wellbeing was affected by vulnerability due to the smallness of the business and the absence of available resources. Simultaneously, a small company context provided a strong social climate and close relationships with employees and customers that strengthened the managers’ wellbeing. The findings suggest that the availability of financial, personnel, and organizational resources varies between small companies of different size, which may have implications for small business managers’ work and wellbeing.

## Introduction

It is well established that working environment affects wellbeing (e.g., Häusser et al., 2010; Niedhammer et al., 2021; Nixon et al., 2011; Van der Doef & Maes, 1999). Managers’ wellbeing has explicit implications for their leadership behaviours (Harms et al., 2017; Joseph et al., 2015; Kaluza et al., 2020), as well as for employees’ wellbeing (Skakon et al., 2010). Managers’ wellbeing is especially crucial in small companies (Lechat & Torrès, 2017), due to their central role in the business (O’Gorman et al., 2005). Small business managers’ wellbeing also has consequences for their own performance, decision making, and action (Cocker et al., 2013; Fernet et al., 2016), as well as for business success and survival (Dijkhuizen et al., 2018; Fernet et al., 2016; Gorgievski et al., 2010; Hessels et al., 2018). Small companies play a crucial role in national economies and employment (Barbosa et al., 2019; Owalla et al., 2022; Visentin et al., 2020). Therefore, it is important to understand the conditions, factors, and circumstances in the work environment of managers in small businesses that can influence these managers’ wellbeing.

Wellbeing is a broad concept including many dimensions (Diener, 1984; Warr & Nielsen, 2018). This study focuses on subjective well-being which is defined as an individual’s assessment of his/her life based on his/her unique perspectives (Diener et al., 2018), beyond physical and psychological health (Danna & Griffin, 1999; Sonnentag, 2015). This concept encompasses evaluations of different aspects of a person’s life, work, health, relationships and sense of purpose as well as affective states, including both positive (such as job satisfaction, engagement, and positive emotions) and negative (like job stress, and negative emotions) (Diener & Ryan, 2009).

This study examines factors that are essential for the subjective wellbeing of small business managers through the lens of the job demands—resources (JD-R) model (Bakker & Demerouti, 2007, 2017; Demerouti et al., 2001). The model explains occupational wellbeing through job characteristics and conditions, classified as either job demands or job resources (Demerouti et al., 2001). It builds on theories such as the demand—control–support model (Johnson & Hall, 1988; Karasek & Theorell, 1990) and the effort—reward imbalance model (Siegrist, 1996, 2002), and assumes that work situations with high job demands and low job resources will increase work-related strain and lower work engagement, thus affecting individuals’ health (Bakker et al., 2004). In general, increased job demands constitute a negative impact on wellbeing and increased job resources a positive one (Crawford et al., 2010; Häusser et al., 2010; Sanz-Vergel et al., 2014). Job demands include aspects of the job that need investment of physical and psychological efforts and entail physiological and psychological costs (Demerouti et al., 2001). Resources facilitate achievement of work objectives, reduce demands and their related physiological and psychological costs, and/or contribute to personal growth and development (Bakker et al., 2004). There are resources on the organizational, interpersonal, and individual level (Bakker & Demerouti, 2007).

Previous empirical research illustrates the job of a manager as a stressful one characterized by fragmentation, complexity, change, and uncertainty (Ganster, 2005; Mintzberg, 1973; Quick et al., 2000). Managers work long hours, and carry out a heavy workload at a rapid pace (Carlson, 1991; Ganster, 2005; Mintzberg, 1973; Quick et al., 2000; Tengblad, 2006). However, despite high job demands, managers also have high levels of control, decision authority, and autonomy as job resources (Bernin & Theorell, 2001; Li et al., 2018; Nyberg et al., 2015).

Entrepreneurs, which in our context means manager-owners, have also been shown to have work that is highly demanding and stressful (Mäkiniemi et al., 2021; Omrane et al., 2018; Sardeshmukh et al., 2021; Stephan, 2018). The very nature of entrepreneurs’ work, including tasks, responsibilities, and challenges related to owning and operating a business, can in itself lead to high strain and burnout (Fernet et al., 2016; Hessels et al., 2018) and to the likelihood of other stressors such as work-life conflict, role ambiguity, and financial pressure (Cocker et al., 2013). Common features of entrepreneurs’ work are long working hours, multiple roles, dealing with uncertainty and an unpredictable environment, high time and energy investment, loneliness, and low support (Cocker et al., 2013; Fernet et al., 2016; Mäkiniemi et al., 2021; Omrane et al., 2018; Sardeshmukh et al., 2021; Visentin et al., 2020). However, despite having highly stressful work, business owners enjoy high satisfaction (Mäkiniemi et al., 2021) as well as great autonomy and control over their work (De Mol et al., 2018; Visentin et al., 2020). Moreover, running a small business is experienced as being rewarding and meaningful (Visentin et al., 2020).

Studies in recent years have pointed to an array of factors that predict entrepreneurs’ wellbeing. Recent reviews (Mäkiniemi et al., 2021; Stephan, 2018) of available research classify antecedents of entrepreneurs’ wellbeing on levels related to job factors (e.g., high workload, role ambiguity, autonomy, flexibility, significance), social factors (e.g., support from family and peers, feedback from clients, work-family conflict, customer and employee conflicts), firm-related factors (income, subjective financial success, firm size, financial problems, job insecurity and uncertainty), personal factors (e.g., personality traits, human capital, values, and motivation) and societal factors. Lechat and Torrès (2017) found that the most frequently experienced stressor for owner-managers of SMEs was overwork, followed by pressure from competition and loss of clients; and that client satisfaction, strategy success, and good social climate were the most positive factors.

However, research has not sufficiently addressed the occupational determinants of small business managers’ wellbeing (Lechat & Torrès, 2017). This group might differ from the general population of managers due to the specificity of small businesses (Torrès & Julien, 2005) and manager-owners’ different roles as entrepreneur, operative manager, and even professional worker. Most studies focusing on antecedents of managers’ wellbeing have been carried out in the context of public organizations (e.g., Asplund et al., 2022; Björklund et al., 2013; Lindholm, 2006; Skagert et al., 2012) or large companies (Lundqvist et al., 2012; Nyberg et al., 2015), and small firms have been overlooked.

Studies that address entrepreneurs’ working conditions and wellbeing (e.g., Hessels et al., 2018; Mäkiniemi et al., 2021; Stephan, 2018), stemming from the domain of entrepreneurship, are of relevance for the population of managers in small companies. However, research in this field has been criticized for treating a broad population of entrepreneurs as a single homogenous group (Barbosa et al., 2019; Owalla et al., 2022) and for not paying sufficient attention to the organizational contexts in which entrepreneurs operate (Hessels et al., 2018). The population of entrepreneurs includes different types; for instance, self-employed entrepreneurs with or without employees, and owner-managers in small or medium companies (Hessels et al., 2018; Mäkiniemi et al., 2021). Different types of entrepreneurship, sizes of firms, years in business, and other characteristics can lead to variations in organizational structure and also in economic, human, and technological resources (Barbosa et al., 2019). These, in turn, may affect entrepreneurs’ work, working conditions, and wellbeing, which justifies their incorporation in research studies (Hessels et al., 2018; Mäkiniemi et al., 2021).

Overall, given the importance of small businesses for society, and the significance of managers’ wellbeing for employee health, leadership, and business performance, more knowledge is needed on the sources of managers’ wellbeing in small companies. Further, existing research on working conditions and health has been dominated by quantitative and variable-centred research methodologies (Corin, 2016; Mayer & Boness, 2011). Thus, there is a need for qualitative studies that can provide a better understanding of the occupational conditions associated with managers’ wellbeing in the context of small companies. This study therefore aimed to explore which factors within the small business context were perceived by managers to hinder or enable their wellbeing.

## Materials and methods

### Study design and sample

A qualitative approach was employed, with data drawn from 20 interviews with managers from 12 small companies. The selection of the companies took place in the context of a regional project, “Successful Companies in Gästrikland” (SCiG), that awards annual honours to successful companies in Gästrikland, a region in mid-Sweden. Companies at the top of the ranking list (made on objective assessment of profitable growth) are nominated for the award. A full description of the selection process can be found elsewhere (Ahmadi et al., 2021).

Small companies that had no more than 50 employees, had been operating since at least 2008, and that were on the project’s nomination list during 2008–2019 were selected. Interviews were conducted until data saturation was reached, which amounted to 20 managers from a total of 12 companies. Nine of the companies had been nominated for the award more than seven times during 2008–2019, indicating a sustainable profitable growth in a longer time-perspective. The three other companies had only once been among the highest ranked companies, indicating a shorter profitable growth during recent years.

The first author approached the CEOs of the selected companies by email, inviting them to participate in the study and informing them about the study purpose, the data collection procedure, and how the data would be treated. This was followed by telephone contact to confirm participation in the study and to discuss practical arrangements for data collection. The CEOs informed their lower managers about the study and asked whether they were willing to take part in the interviews.

At the time of the interviews, the companies had been in business for 12–51 years and had 4–46 employees. They represented the following branches of business: sales (*n* = 5), manufacturing (*n* = 4), technical consulting (*n* = 2), and transport (*n* = 1). The participants consisted of 12 executive directors (CEOs; ten of them owner-managers), and eight managers of lower level. Both categories of managers were included in the study due to their potential impact on employee wellbeing and consequently on the effectiveness of business, something that to our knowledge has been overlooked in previous studies on small businesses (cf. Dijkhuizen et al., 2016; Lechat & Torrès, 2017). They comprised 18 men and two women, were aged between 29 and 66 years old, and had managerial experience ranging from 2.5 years to 29 years (Table I).

**Table I.** Sociodemographic characteristics of the participants.

	N=20
*Position*	
CEO	12
Lower manager	8
*Sex*	
Male	18
Female	2
*Education*	
Secondary education and similar	16
University education	4
	*mean*
Age, years	47
Manager experience, years	14

### Data collection procedure

The interviews were performed in 2020, using a semi-structured interview guide that included several predefined topics and open-ended questions enabling follow-up and clarification through probing questions (Kvale & Brinkmann, 2009). This allowed respondents to unfold their perspectives and experiences according to their own ways of structuring and framing the response. The main themes in the interview guide concerned managers’ wellbeing, working environment, and work-related factors influencing their wellbeing. Examples of the questions are: “Is there something in your everyday work that makes you feel good?”, “What is the most important factor for you to feel good at work?”, and “What in your work makes you not feel good?”, with the subsequent questions challenging them to elaborate on and exemplify their answers. No definitions of wellbeing were provided to the respondents.

The first author conducted the interviews in the forms and venues of the participants’ choice; either face-to-face in the companies’ premises (*n* = 18) or remotely using the Zoom video conferencing service (*n* = 2). Interview duration was between 60 and 90 minutes. Saturation was observed after 20 interviews, since no new aspects were emerging in relation to the experiences and perceptions of factors influencing managers’ wellbeing. All interviews were audio recorded with the participants’ consent, and were transcribed verbatim by a professional transcriber (*n* = 17) and by the first author (*n* = 3).

### Data analysis

Qualitative content analysis (Elo & Kyngäs, 2008; Graneheim & Lundman, 2004; Hsieh & Shannon, 2005) was used to analyse the data, allowing the study phenomena to be described through the identification of core meanings among the respondents’ answers. An inductive approach was applied in the analysis due to the limited nature of previous research on this group and to avoid delimiting the analysis to predefined categories based on existing quantitative studies. The transcribed material was read several times to achieve immersion. The texts were then transferred to version 9 of ATLAS.ti for Windows (ATLAS.ti Scientific Software Development GmbH, www.atlasti.com), a computer-assisted qualitative data analysis software package, for the analysis. Texts were divided into meaning units corresponding to the aim of the study. The length of these meaning units varied from several sentences to a few words. The meaning units were then coded by assigning each one a label describing its content and the core meaning. The initial codes were sorted by comparing for similarities and differences, and then abstracted to overarching categories that showed patterns in the material. Categories answering the question “what” (Morse, 2008) and describing the group of codes that they comprised (Lindgren et al., 2020) were used in the analysis.

The process of coding, sorting, and abstracting involved moving back and forth between meaning units, codes, and categories, and between parts and the whole, which entailed several steps of reworking. This analysis resulted in a category list describing the participants’ perceptions and experiences related to the study’s aim. In the Results section below, the identified categories are presented in a category matrix, and then described in detail and illustrated with quotations from the participants. The analysis of the data was performed close to the manifest content in the original text, with a low degree of abstraction and interpretation (Lindgren et al., 2020). The analysis was thus limited to the formulation of categories on a descriptive level (Lindgren et al., 2020), which suited the purposes of this study. The first author carried out the primary analysis and discussed the process of sorting and abstraction with the second author, and then all the authors discussed and reviewed the categories.

### Ethical considerations

The study was approved by the Swedish Ethical Review Authority (approval number 2019–00314) and performed in accordance with the principles of the Declaration of Helsinki. All participants were given information regarding the purpose of the study, the voluntary nature of participation, the principles of anonymity and confidentiality, and their right to withdraw from the study at any point without need to give a reason. Informed consent was obtained from all participants before their interview.

## Results

This section presents results related to factors which managers perceived as important for their wellbeing. Respondents’ answers were clustered around five major categories: daily managerial work, achieving results, social factors, organizational factors, and individual factors. Answers related to each category were classified in terms of hindering factors (impeding managers’ wellbeing) and enabling factors (contributing to managers’ wellbeing). The categories are summarized in Table II and described in detail with corresponding quotations in the following text.

**Table II.** Factors that managers experienced as hindering or enabling their own wellbeing.

Category	Hindering factors	Enabling factors
Daily managerial work	High workload and time pressure Unforeseen events and difficulties Fragmentation and disruption Role structure and role conflict Responsibility over company and employees	Manageable workload, control of own work, freedom to steer working time Smooth operation Stimulating and varied work Personal and company development, having a key role in the company Significance and meaningfulness (appreciation of the work, the product, running business and serving others, doing meaningful work)
Achieving results	Failure to achieve results and meet goals individually and collectively Failure to meet deadlines or provide good quality, customer dissatisfaction Poor performance by self, employees and company	Achieving good results individually and collectively Succeeding in providing good quality, successful sales, satisfied customers/positive feedback from customers, receiving new orders Good performance by managers, employees, and company
Social factors	Personnel-related problems (conflicts, unsatisfied employees) Employees feeling unwell both physically and psychologically Lack of social support	Employees’ satisfaction and wellbeing Interaction and good relationships with employees Positive and joyful atmosphere at the workplace Contacts and interactions with customers Social support internally (employees, steering boards, co-owners, other levels of management) and externally (customers, family)
Organisational factors	Vulnerability inherent in small businesses (insufficient financial and personnel resources)	Sufficient and reliable financial and personnel resources Well-functioning organisation (clear structure and roles, management spread at different levels), Technical and administrative staff, digital support systems
Individual factors	Home-work conflict, family situation, and private problems	Accepting disturbance, unforeseen incidents, and missing deadlines as a part of managerial job, changing attitude to demands. Handling stressors through planning, prioritising, focusing on doing work step-by-step Managerial work experience, positive personality and positive attitude, age

### Daily managerial work

#### Hindering factors in daily managerial work

*High workload and time pressure* were pointed out as stressing factors. Most managers reported working between 40 and 60 hours per week, and considered their workload high but manageable. They experienced stress when they had to achieve too many different things in too little time, were lagging behind deadlines, were working overtime, were not able to concentrate on their tasks, and had the feeling of not doing a good job.

It’s stressful when you feel the pressure, that you have to work more to catch up. Then being a manager isn’t fun any more… Yes, it’s lack of time, more tasks than you really have time for, that you’d like to concentrate on… you think you’re not doing the job you’d like to do. (IP 6, CEO)

However, managers did not think that these stress-filled situations affected them substantially, since such situations were confined to limited time periods and they had the possibility to recover afterwards.

*Unforeseen incidents, difficulties, and complications* in the work were experienced as stress-inducing. Examples of such factors were when things did not go as planned, machines broke, people got sick, subcontractors did not deliver on time, and several urgent orders arrived simultaneously. As a result, managers needed to put extra effort into solving the problems, finding replacements for sick employees, and rescheduling work, which disrupted the other work they had planned to do that day. Postponing the delivery of company products due to unforeseen events could lead to deterioration of client relations and jeopardize the company’s reputation.

When subcontractors don’t deliver, I get so annoyed that I could snap. When we’ve sold the product to a customer after an agreement with our subcontractors, and then we have to admit that we can’t deliver in time… then it’s hugely stressful. (IP 16, lower manager)

*Disturbance and interruption* of the work was described by some managers as stressful. The managers felt that their work became fragmented, and that they could not perform their tasks as planned, which was especially frustrating in combination with a high workload.

When you have a lot to do, and employees come and need help with something. These unplanned activities, they stress me a lot. (IP 11, CEO)

*Responsibility for the business and staff* was also referred to as demanding. Several owner-managers described constantly having concerns and thoughts about the company’s current situation and future development; they needed to be alert, to tackle fluctuations in the company finances, and to adjust for changes in the external environment.

I go home and think about the company… the company’s in my head all the time. (IP 18, CEO)

It’s not a bed of roses [running a business]. There are challenges all the time. You can never let it go completely¸ you sit there on the weekends thinking… it’s with you all the time … (IP 4, CEO)

One CEO explained how challenging it was to have the main responsibility for the employees’ income and for providing them with secure employment.

You are a business owner. And that means you’re still one when you get home. When there are vacation periods, it’s my responsibility to make sure it works. The employees book their vacation period and we’ll see if there will be any time left for me. You have to put yourself in second place. The company comes first. The employees come first. You are always an entrepreneur with responsibilities towards your employees, to pay the wages … . (IP 15, CEO)

Several managers, however, described this situation as a natural part of running a business and nothing extraordinary.

*Role conflict and unclear management structure* were other hindering factors experienced by some managers. For instance, one lower manager felt dissatisfaction over not having the mandate and trust to make desirable changes, especially when he and the owner-managers had different views about the company’s development. In addition, some managers described an unclear division of roles and responsibilities between the owner-managers who were both operational in the company and part of the management group, and sometimes also board members.

#### Enabling factors in daily managerial work

*A manageable workload, a feeling of being in control at work, and the freedom to steer their own working time* were pointed out by the respondents as positive factors for their wellbeing.

The freedom … that I can control myself and others… the freedom to be able to go away for a while during the day or do what I need to do … (IP 20, lower manager)

*Smooth and trouble-free operation*, when everything went as planned without major problems, was experienced by the managers as a favourable condition. However, the managers expressed that needing to tackle challenges made their work more *stimulating* and contributed to their job satisfaction.

*Variation* in work, when the everyday work was not monotonous, many things were happening, and tasks were varied, was also highlighted as an important factor contributing to work satisfaction.

The variety of tasks … not always doing things I’ve done before … to be able to influence the organisation. To be involved in setting things in motion and helping employees to grow. I find the whole combination of these things to be stimulating. (IP 12, lower manager)

*Personal and company development* was another source of job satisfaction and wellbeing. The managers found it uplifting to have *a key role in the firm*, to be able to *contribute to the development of the company, the employees, and the products*, and to experience *their own development and growth*.

Finally, *significance and meaningfulness* of the work were also mentioned as enabling factors. Several respondents enjoyed what they were doing (e.g., building cars, selling products) or believed that they were creating meaningful things (e.g., products and services that contributed to the community, to better health, to the environment), while others felt good when they could help others (e.g., by solving customers’ problems and contributing to employees’ wellbeing).

The best thing about my job is that I have an opportunity to be the spider in the web, be a little involved in everything… have a key role in the company… to be there when things happen. (IP 14, lower manager)

Having the opportunity to be part of creating a workplace where people grow and feel good, that’s the most fun. (IP 10, CEO)

### Achieving results

#### Hindering factors related to achieving results

The managers maintained that their wellbeing was affected negatively when the company could not meet deadlines, could not provide a high service level and good quality, failed to satisfy customers, or when they themselves could not meet their own work goals.

#### Enabling factors related to achieving results

Factors contributing to the managers’ wellbeing and overall satisfaction with their situation included a feeling of satisfaction with their own performance, feeling that they were doing a good job, accomplishing their tasks, and receiving appreciation. Similar effects came from the company’s good performance, employees’ success in delivering good products and services, achieving good sales rates, and customers being satisfied with the company. However, achievement of results could both hinder and enable wellbeing, as exemplified in the quotation below.

I’m happy when we succeed, when the staff are happy, when we don’t have a lot of problems, and when customers thank us for being good at what we do. A good day is when everything is going well, and you go home in the evening and think “Now we’ve succeeded in what we set out to do,” then you feel good, then you get a little kick, God, it’s nice. Some days when you go home and know that we only managed to do half of what we were supposed to do, you feel bad yourself. Then you take that with you; it’s a bad feeling you take with you when you go home. (IP 8, CEO)

### Social factors

#### Hindering social factors

Personnel-related problems such as conflicts, unsatisfied employees, negative attitudes, and employees not feeling good were considered particularly demanding, draining the managers’ energy and reducing their motivation. Some interviewees explained that these problems made them dissatisfied with their own job, the results, and the company. Several stated that they lacked knowledge of how to handle these kinds of problems. Furthermore, they felt worse psychologically when their employees did not feel good. Some explained this in relation to negative effects on productivity and meeting deadlines, but they were also concerned about the employees as individuals, due to their close relations.

When there are conflicts … when the staff don’t feel good … then of course it affects me too. I feel good when everything is fine and when the employees feel good. (IP 9, lower manager)

#### Enabling social factors

When the employees were satisfied and felt good, this contributed to the managers’ work satisfaction and wellbeing. The managers also highlighted that a positive and joyful atmosphere at the workplace, a culture of helping each other and having good relationships, and interactions with and among employees made them feel better and appreciate their work.

For me, it’s important to be able to meet the staff, see how they’re doing, hear if they have any problems and if I can be involved and help with something … This makes me feel good. I think they also benefit from this … (IP 20, CEO)

Several managers expressed that they liked working with people and appreciated having interpersonal contacts in the process of running the business, both with customers and with employees, and that these contributed to their own job satisfaction and wellbeing.

The managers stated that support was important, both from outside the company (e.g., from external board members and mentors from larger companies) and from within the company (e.g., from employees, management teams, and other co-owners). CEOs emphasized the lower managers’ role in facilitating their own work; and lower managers, in turn, valued the support from CEOs through consulting, advice, and discussing problems and work issues. Several CEOs pointed out that support from their families was very important for their work, either in terms of understanding and accepting their working situation, by temporarily taking a more active role in the household and care of children, and even by helping in the company.

### Organisational factors

Responses in this category differed between the smallest companies (4–6 employees, with a shorter growth period) and the largest ones (10–46 employees, with a longer growth period).

#### Hindering organisational factors

Managers of the smallest companies talked about the vulnerability of their businesses due to limited financial and personnel resources. One manager explained that when an employee was sick, either somebody else needed to step in and work overtime, or they would have to alter the overall planning, postpone deliveries, and thus increase the risk for the cash flow. He described this as a difficult balancing act between achieving results and not overloading the staff. Often it was difficult to recruit a short-term replacement from outside the company, due to the specificity of job skills and the need for long training.

If we [the company] are only three or four people and one is sick, then the others must step in and do that job. Then there could be problems… I don’t want to burden them [the employees] fully either, because I know that if I put a lot of stuff that needs to be done on someone, then that person gets sick too and we have nobody to do those things anyway … (IP 20, CEO)

We’re so pressured at every position we’re in that when someone is absent from something there will be chaos and panic for everyone … (IP 19, lower manager)

Managers of the smallest companies also said that demands related to workload, work pace, and unforeseen events were intensified when the manager worked part of their time in management and part of their time performing professional operational tasks. This situation arose since these small businesses could not afford to have full-time managers. One CEO described having to jump in and do emergency jobs when somebody got sick, implying that his own managerial and administrative work was postponed to after the regular working hours. Another said that despite his intentions to delegate a part of his own responsibilities, he had to do everything himself since there were too few employees at his company.

#### Enabling organisational factors

Some managers of the largest of the small companies considered strong financial and personnel resources as facilitating factors. They mentioned that a good financial situation provided a stable platform to stand on, acted as a buffer for difficult times, and created psychological security for the manager and employees. The managers felt that sufficient personnel resources were very important for running a business (for instance, by increasing the possibility of finding a replacement when somebody was sick) and for ensuring a manageable workload for employees and the CEO.

The managers explained that having a structure of leadership at different levels, with lower managers and supervisors, enabled effective operative management, control over the situation, and the possibility for problems to be solved without requiring the CEO’s involvement in every issue. They also considered that professional staff within finance and administration could provide support and relieve managers from these tasks.

The managers noted that a well-functioning and structured organization with defined roles, planning, and routines could facilitate their managerial work. These aspects provided security by giving them better control over the situation and enabling smooth operations with as few errors as possible.

… it makes it easier when we can plan our work, get structure and order. We make the processes run smoothly. (IP 13, CEO)

Digital support systems also enabled control over processes within the company such as finance, production, warehouse, and procurements, which according to the managers facilitated their everyday work and reduced inefficiencies and mistakes.

### Individual factors

#### Hindering factors

The managers explained that high workload and time pressure affected their work-life balance negatively and made their work more stressful, especially when combined with private arrangements or problems. Some respondents also mentioned that their family situation affected how they perceived their workload.

…sometimes during the weeks I have my son with me, I’m very limited, especially when he’s sick or if I have to adjust my working hours to drop him off and pick him up. At the same time, I know I’m already lagging behind with work and meetings, and the employees are waiting for me and need me. It’s clear that the stress becomes more noticeable then. (IP 13, CEO)

#### Enabling factors

The managers described having learnt to handle stress in their work, for instance by better planning, prioritizing, and focusing on doing the work step-by-step. They had also learnt how to cope better with stress, thanks to their changed attitude towards the demands and putting less pressure on themselves. For instance, several managers had learnt to accept that disturbances and unforeseen incidents were a part of the managerial work, that things must take time, and that it was fine not to accomplish everything on time and to sometimes refrain from accepting new orders. Some managers stated that they had become better at coping with stress as they got older, while others thought that their increased working experience had made it easier to change their attitude and endure.

… it’s important that you can then feel safe, you know how it was the previous year, so you don’t have to get stressed. You know what’s coming, you’re prepared for it, and with that you can make a better plan. (IP 16, lower manager)

I’ve been doing this for 30 years, it’s like it’s in my DNA. You aren’t involved in so many new incidents. Almost every year looks similar. It’s the weather and the wind that make a difference. (IP 1, CEO)

Several managers expressed that their personality and positive attitude had helped them, since they saw problems as challenges and as a part of their working tasks.

## Discussion

The aim of this study was to explore which factors within the small business context were perceived by managers as hindering or enabling their wellbeing. The results revealed five categories of factors, including both demands and resources: daily managerial work, achieving results, social factors, organizational factors, and personal factors. These five types generally resemble the predictor groups identified in previous research focusing on larger organizations (e.g., Lundqvist et al., 2012) and entrepreneurs (Mäkiniemi et al., 2021; Stephan, 2018). However, the context of small companies adds unique features and nuances to the general picture.

The first category of factors, daily managerial work, concerned the demands and resources embedded in the daily work practices. Demands that were emphasized involved high workload, time pressure, and unplanned incidents while resources involved autonomy, task variation, and the possibility to influence activities in the organization. These results are similar to previous findings (Lundqvist et al., 2012; Mäkiniemi et al., 2021; Stephan, 2018), which may reflect universal traits in managerial work (Mintzberg, 1973; Tengblad, 2006). However, this study also highlighted demands on owner-managers in relation to responsibility for employees’ job security and the company’s survival and development. Another resource, that managers in this study perceived important, was related to meaningfulness and significance of their work manifested in managers’ appreciation of running a business, working with the company’s products, and being able to help and serve others. This suggests that these factors reflect the entrepreneurial dimensions in the work of owner-managers in small organizations caused by their central position in the company, personal investment, and self-identification with the business.

The second category of factors identified in this study, pertaining to achieving results individually and collectively, has not been a frequent focus of previous research (except for a few studies such as Lundqvist et al., 2012). This reveals a distinctly marked difference between the results of this study and previous research. A possible explanation for this divergence in findings may be that achievement of results has often been treated as an outcome (using quantitative methodologies) rather than as an antecedent affecting wellbeing. The present study demonstrates clearly that the achievement of results and the feeling of doing a good job personally and collectively are perceived to be central in the creation of managers’ wellbeing. The importance of this factor may be further accentuated in the context of small businesses, because of the managers’ personal investment of time and money in the business as well as their overall responsibility for the future of the company and their association of the business’s failure with their own (Lechat & Torrès, 2017).

The third category covered the social environment of managers. The findings within this factor confirm previous research on entrepreneurs’ antecedents of wellbeing in relation to conflicts and problems with personnel, the importance of social support, and work-family conflict (Mäkiniemi et al., 2021; Stephan, 2018). However, the results of this study widen the social factors to include dimensions such as positive social climate, interpersonal relations with employees and customers, and employees’ wellbeing. It is likely that these resources are especially important in the context of small businesses, which is characterized by proximity in relationships with employees and customers (Torrès & Julien, 2005). This is in line with the findings of Lechat and Torrès (2017) that good social climate and meeting with employees are among some of the satisfactory factors in entrepreneurial activity. The close interactions between employees and managers in small companies may make shifts in wellbeing more marked. Employees’ low wellbeing and sickness absence are experienced as more tangible and have larger consequences for managers’ work and wellbeing in this context, due to the limited personnel resources. This can make managers of smaller businesses more dependent on employees’ wellbeing as compared to their counterparts at larger companies.

Interestingly, managers in this study did not mention loneliness, which is a stressor commonly attributed to both managers’ (Lundqvist et al., 2018) and entrepreneurs’ work (Fernet et al., 2016; Mäkiniemi et al., 2021; Stephan, 2018). A possible explanation could be that managers in small companies experience a family-like atmosphere at the workplace, where they have daily contact with employees and customers.

The fourth category of factors was related to the availability of financial, personnel, and organizational resources, and the consequent vulnerability of small businesses. Previous research has highlighted financial problems, job insecurity, satisfaction with income, and perceived company success as predictors of managers’ wellbeing at the company level (Mäkiniemi et al., 2021; Stephan, 2018). The findings in this study do not directly point at personal earnings and firms’ financial characteristics as facilitators and hindrances in managerial work and managers’ wellbeing, but rather to the company’s resources.

The present results suggest that the context of small businesses can influence the demands on managers and their resources, but they also point to variability within the context of small companies due to firm size. In contrast to the classical concept of the stable nature of managers’ work across different contexts (Mintzberg, 1973; Tengblad, 2006), Ahmadi et al. (2021) have shown that company size matters for managerial work and that the group of small companies is not homogenous. In the present study, the smallest companies showed vulnerability due to poor resources, which aggravated managers’ working situation, while larger small companies did not experience this vulnerability. These larger companies seemed to have a larger reservoir of resources at the organizational level, which may have helped their managers to withstand stressors. The question of stability and variability of managerial work in the small business context, as well as its implications for organizational health, warrants further investigation.

Regarding the fifth factor category, individual factors, the study findings are in line with previous research (Mäkiniemi et al., 2021; Stephan, 2018) in relation to coping, focus on opportunities, age, entrepreneurial skills, leadership experience, and work-life balance.

Overall, the results of this study identify factors that managers in small businesses experienced as enabling or hindering their wellbeing. When viewed through the lens of the JD-R model (Bakker & Demerouti, 2007, 2017), these can be regarded as resources and demands, respectively. The findings suggest that managers’ work in small businesses is characterized by high demands, and that they need to handle a constant stream of problems and challenges that require investment of time and effort, which together may lead to drained energy, strain, and a negative impact on wellbeing. Conversely, the findings also reveal several work characteristics and conditions that managers of small businesses perceived as valuable resources for their wellbeing. These resources could contribute to motivational process, flourishing, and wellbeing, but may also help managers to alleviate their job demands by better coping (Bakker & Demerouti, 2017). To some extent, the present findings resemble empirical evidence found elsewhere, where managers’ work was characterized by high levels of both demands and resources (Li et al., 2018).

It seems that in the context of small businesses, managers’ demands and resources combine those pertinent to both the managerial and the entrepreneurial work situation. Therefore, it is worth asking what similarities and differences there are between managers’ and entrepreneurs’ working conditions regarding their wellbeing. Traditionally, entrepreneurs’ and managers’ work and working conditions have been compared to the general employed population and non-managers, and not between themselves.

Although many factors identified in this study have been discussed in previous studies, there are several novel findings that can be attributed to the specific context of small businesses and may be less evident in the context of large companies. The findings point out that the availability of resources and the consequent vulnerability of businesses is related to the size and smallness of the companies. Managers’ concern and responsibility for the success of the business, a stable income for the staff, and achievement of results individually and collectively can be further enhanced in this context due to owner-managers’ identification with business and personal investment. Finally, interpersonal relations with employees and customers, and employee wellbeing can also be related to the specificity of small companies, due to the short distance between the manager and the employees, intense everyday contact, and the central role that managers play in operational management.

It is worth mentioning that these resources may also be influenced by leadership behaviours and the structure of the organization. In a recent study, Ahmadi et al. (2022) found that factors that can be affected by managers’ wellbeing include the working climate, work relations, and interaction with employees as well as managers’ own performance and achievement of results. In other words, these factors may be both consequences and antecedents of managers’ wellbeing, and might be influenced both by managers’ behaviours and by the organizational context of the companies.

The results also suggest that the identified factors are interrelated with each other. For instance, the small businesses’ vulnerability seemed to intensify the experience of demands. The success or failure in responding to demands influenced the managers’ sense of achievement. Good social climate and close relationships are also favourable for employees’ wellbeing (Crawford et al., 2010; Nielsen et al., 2017), which in turn is an important factor for managers’ wellbeing. Therefore, it is not plausible to point at a single factor as more important than any other, because the managers’ work situation with its embedded factors should be considered as a whole. However, since the factors seem to be related, it also means that improvement in one might lead to improvement in others as well.

Interestingly, we can see that several demands and resources in the context of small businesses mirror each other. Some of the demands identified in this study were in fact the opposite of the corresponding resources. Referring to the same factors as both demands and resources may reflect the absence/presence and even intensity of the basic factor in cases such as employee wellbeing, social support, and company performance.

Some other factors were perceived to both increase and decrease the managers’ wellbeing. For example, unforeseen events were thought of as both stressful and stimulating, and were thus classified as both demands and resources. Even heavy workload appeared to be both challenging and manageable. This can be related to previous research which divides demands into two types: challenges and hindrances (Crawford et al., 2010; Lepine et al., 2005). Hindering demands lead to exhaustion and lower engagement, while challenging demands foster engagement without having a negative effect on wellbeing (Crawford et al., 2010; Lepine et al., 2005). It is therefore important to know when and why demands are appraised as hindrances or challenges (Bakker & Demerouti, 2017; Crawford et al., 2010). It is plausible that when demands become more manageable, they can be experienced more as challenges. If these challenges are handled successfully, managers can experience a sense of achievement, which, as shown in this study, is an important resource for their wellbeing.

The findings also indicate that individual and organizational resources may enhance managers’ ability to cope with their job demands. Central dimensions in this respect are managers’ professional experience (making work more predictable) and accepting problems and interruptions as inherent to managerial work. It is obvious that unforeseen events, problems, and difficulties in planning the company operations are inherent to managerial work, and cannot be completely avoided. However, larger organizational resources might increase managers’ preparedness and capacity to tackle emerging problems, reduce the degree of uncertainty, and thus reduce the strength of the demands and increase the sense of manageability. Nevertheless, it is plausible to assume that the intensity of a demand factor (e.g., having a very high workload) could also influence whether the demand is perceived as a hindrance or a challenge. If the levels of demands remain chronically high, this may lead to negative consequences for managers’ wellbeing in the longer term (Bakker & Demerouti, 2017). It is therefore important to pay attention to the intensity of demands as well as the availability of sufficient resources to support managers.

There are some indications in the findings that the intensity of demands such as workload and work pace may often fluctuate for small business managers. This points to temporal variations in the strength of daily, weekly, or seasonal demands. In the same way, there might be temporal variations in other demands, such as unforeseen incidents, and troubles with personnel and customers alike. Since handling high demands requires energy (Bakker & Demerouti, 2007) and drains managers’ resources, this may bring short-term variations in managers’ wellbeing. This finding is in agreement with existing empirical evidence that highlights daily fluctuations in job demands and resources (Bakker, 2014) and fluctuations in managers’ wellbeing (Ahmadi et al., 2022; Barnes et al., 2015).

## Strengths and limitations

This study has several strengths. Firstly, it responds to the calls to enhance knowledge on sources of small business managers’ wellbeing while taking the social and organizational context into consideration (Cocker et al., 2013; Stephan, 2018; Visentin et al., 2020). Secondly, its findings enhance knowledge of factors that managers themselves experience as important for their wellbeing in the specific setting of small businesses. Thirdly, the study highlights that company context shapes managers’ working situation and consequently their wellbeing.

Fourthly, it has a practical implication, as the dissemination activities of the SCiG project will lead to managers becoming informed about both the availability of the resources in their own work situation for promoting their wellbeing, and the demands that are especially detrimental for this. Managers will thus be helped to sustain their wellbeing and to conduct a productive work life with positive consequences for their performance, leadership, employee health, and organizational effectiveness. In addition, managers may become more attentive to analysing their own working conditions, and hence to working proactively to enhance their own job resources and reduce the demands. More specifically, regarding factors that are characteristic of small businesses, this study draws managers’ attention to the importance of fostering an open and positive atmosphere at the workplace, investing in regular contacts and interactions with employees, and promoting employee health as favourable preconditions for their own wellbeing. From a societal perspective, the study provides insights that may be of importance to policy makers on the need to create support structures and programmes for small businesses to mitigate the vulnerabilities faced by start-ups and small businesses, and thereby improve managers’ working conditions and, consequently, their own and their organizations’ wellbeing.

Despite the abovementioned strengths, the study has some limitations. For instance, due to the nature of the sample (award-winning companies) and the sample size, it was not possible to investigate variations in companies’ or managers’ characteristics; for example, gender. Most of the managers in these companies were men, which reflects the sociodemographic features of the region, with traditionally male-dominated businesses.

Another limitation is that the COVID-19 pandemic limited the possibility of reaching out to participants and might have diminished managers’ willingness to participate. This could have affected the composition of the sample as well as the results of the study.

The finding of variations in the organizational resources that support these small business managers, according to company size, needs to be further investigated to gain a more in-depth understanding of the differences between the smallest and the largest of the small firms. In this study, we have drawn an immediate picture of managers’ working conditions as currently experienced by them, and have not focused on the changes in the managers’ work and wellbeing related to the dynamic nature of small businesses. This is also something which can be addressed in future studies.

In general, the companies included in the study had experienced a steady profitable growth over several years, and hence were mature companies that had successfully survived over a longer period and adapted to the difficulties. Nonetheless, it is possible that the participants of this study had more positive experiences as compared to managers from companies that were not thriving at the time of the interview. Also, the gender imbalance in the sample (with more men than women) might have affected the results assuming that female managers potentially may experience discrimination or higher degrees of work-family conflict. Therefore, the results of this study cannot be generalized to the whole group of small companies, but primarily to the types of organizations similar to those included in the studied sample. Future research is needed to investigate the working conditions of managers in companies that are at different stages of growth, including start-ups, companies in active growth, and mature businesses, as well as working conditions of female managers in small firms.

Finally, another limitation might be that prior to the interviews, no definition of wellbeing was provided the respondents. Therefore, it is possible that they might have referred to different aspects of well-being when describing the factors that they perceived as influencing their own wellbeing.

## Conclusions

This study identified five categories of factors that managers in small businesses experienced as enhancing or hindering their personal wellbeing. These included demands and resources in the daily managerial work, achievement of results, social factors, organizational factors, and individual factors. Although these categories are generally in line with previous research, the specific context of managerial work in small companies also involves some unique factors. For instance, the small company managers’ wellbeing was affected by vulnerability due to the smallness of the business and the absence of available resources. At the same time, a small company context provided a stronger social climate and close relationships with employees and customers that strengthened the managers’ wellbeing. The results also suggest that the availability of financial, personnel, and organizational resources varies between small companies of different size, which may have implications for small business managers’ work and wellbeing.

## Data Availability

The data presented in this study are available on request from the corresponding author. The data are not publicly available owing to restrictions in the ethical approval for this study.

## References

[cit0001] Ahmadi, E., Lundqvist, D., Bergström, G., & Macassa, G. (2022). Managers’ and employees’ experiences of how managers’ wellbeing impacts their leadership behaviours in Swedish small businesses. *Work*, 75(1), 97–14. Advance online publication 10.3233/WOR-220159 PMC10200148 36591688

[cit0002] Ahmadi, E., Macassa, G., & Larsson, J. (2021). Managers’ work and behaviour patterns in profitable growth SMEs. *Small Business Economics*, 57(2), 849–863. 10.1007/s11187-020-00386-0

[cit0003] Asplund, S., Åhlin, J., Åström, S., & Lindgren, B.-M. (2022). Experiences of being a manager in the municipal sector in rural northern Sweden. *International Journal of Qualitative Studies on Health and Well-Being*, 17(1), 2139346. 10.1080/17482631.2022.2139346 36282243 PMC9621278

[cit0004] Bakker, A. (2014). Daily fluctuations in work engagement. *European Psychologist*, 19(4), 227–236. 10.1027/1016-9040/a000160

[cit0005] Bakker, A., & Demerouti, E. (2007). The job demands‐resources model: State of the art. *Journal of Managerial Psychology*, 22(3), 309–328. 10.1108/02683940710733115

[cit0006] Bakker, A., & Demerouti, E. (2017). Job demands–resources theory: Taking stock and looking forward. *Journal of Occupational Health Psychology*, 22(3), 273–285. 10.1037/ocp0000056 27732008

[cit0007] Bakker, A., Demerouti, E., & Verbeke, W. (2004). Using the job demands-resources model to predict burnout and performance. *Human Resource Management*, 43(1), 83–104. 10.1002/hrm.20004

[cit0008] Barbosa, C., Azevedo, R., & Rodrigues, M. A. (2019). Occupational safety and health performance indicators in SMEs: A literature review. *Work*, 64(2), 217–227. 10.3233/WOR-192988 31524191

[cit0009] Barnes, C., Lucianetti, L., Bhave, D. P., & Christian, M. S. (2015). “You wouldn’t like me when I’m sleepy”: Leaders’ sleep, daily abusive supervision, and work unit engagement. *Academy of Management Journal*, 58(5), 1419–1437. 10.5465/amj.2013.1063

[cit0010] Bernin, P., & Theorell, T. (2001). Demand–control–support among female and male managers in eight Swedish companies. *Stress and Health*, 17(4), 231–243. 10.1002/smi.905

[cit0011] Björklund, C., Lohela-Karlsson, M., Jensen, I., & Bergström, G. (2013). Hierarchies of health: Health and work-related stress of managers in municipalities and county councils in Sweden. *Journal of Occupational and Environmental Medicine*, 55(7), 752–760. 10.1097/JOM.0b013e318295681c 23787564

[cit0012] Carlson, S. (1991). *Executive Behaviour*. Reprinted with Contribution by Henry Mintzberg and Rosemary Stewart. https://uu.diva-portal.org/smash/record.jsf?pid=diva2:109085

[cit0013] Cocker, F., Martin, A., Scott, J., Venn, A., & Sanderson, K. (2013). Psychological distress, related work attendance, and productivity loss in small-to-medium enterprise owner/managers. *International Journal of Environmental Research and Public Health*, 10(10), 5062–5082. 10.3390/ijerph10105062 24132134 PMC3823320

[cit0014] Corin, L. (2016). Job demands, job resources, and consequences for managerial sustainability in the public sector – a contextual approach. https://gupea.ub.gu.se/handle/2077/42312

[cit0015] Crawford, E. R., LePine, J. A., & Rich, B. L. (2010). Linking job demands and resources to employee engagement and burnout: A theoretical extension and meta-analytic test. *Journal of Applied Psychology*, 95(5), 834–848. 10.1037/a0019364 20836586

[cit0016] Danna, K., & Griffin, R. W. (1999). Health and well-being in the workplace: A review and synthesis of the literature. *Journal of Management*, 25(3), 357–384. 10.1177/014920639902500305

[cit0017] Demerouti, E., Bakker, A., Nachreiner, F., & Schaufeli, W. B. (2001). The job demands-resources model of burnout. *Journal of Applied Psychology*, 86(3), 499–512. 10.1037/0021-9010.86.3.499 11419809

[cit0018] De Mol, E., Ho, V. T., & Pollack, J. M. (2018). Predicting entrepreneurial burnout in a moderated mediated model of job fit. *Journal of Small Business Management*, 56(3), 392–411. 10.1111/jsbm.12275

[cit0019] Diener, E. (1984). Subjective well-being. *Psychological Bulletin*, 95(3), 542–575. 10.1037/0033-2909.95.3.542 6399758

[cit0020] Diener, E., Lucas, R. E., Oishi, S., Hall, N., & Donnellan, M. B. (2018). Advances and open questions in the Science of subjective well-being. *Collabra: Psychology*, 4(1), 15. 10.1525/collabra.115 30637366 PMC6329388

[cit0021] Diener, E., & Ryan, K. (2009). Subjective well-being: A general overview. *South African Journal of Psychology*, 39(4), 391–406. 10.1177/008124630903900402

[cit0022] Dijkhuizen, J., Gorgievski, M., van Veldhoven, M., & Schalk, R. (2016). Feeling successful as an entrepreneur: A job demands — resources approach. *International Entrepreneurship & Management Journal*, 12(2), 555–573. 10.1007/s11365-014-0354-z

[cit0023] Dijkhuizen, J., Gorgievski, M., van Veldhoven, M., & Schalk, R. (2018). Well-being, personal success and business performance among entrepreneurs: A two-wave study. *Journal of Happiness Studies*, 19(8), 2187. 10.1007/s10902-017-9914-6

[cit0024] Elo, S., & Kyngäs, H. (2008). The qualitative content analysis process. *Journal of Advanced Nursing*, 62(1), 107–115. 10.1111/j.1365-2648.2007.04569.x 18352969

[cit0025] Fernet, C., Torrès, O., Austin, S., & St-Pierre, J. (2016). The psychological costs of owning and managing an SME: Linking job stressors, occupational loneliness, entrepreneurial orientation, and burnout. *Burnout Research*, 3(2), 45–53. 10.1016/j.burn.2016.03.002

[cit0026] Ganster, D. C. (2005). Executive job demands: Suggestions from a stress and decision-making perspective. *Academy of Management Review*, 30(3), 492–502. 10.5465/amr.2005.17293366

[cit0027] Gorgievski, M. J., Bakker, A., Schaufeli, W. B., van der Veen, H. B., & Giesen, C. W. M. (2010). Financial problems and psychological distress: Investigating reciprocal effects among business owners. *Journal of Occupational and Organizational Psychology*, 83(2), 513–530. 10.1348/096317909X434032

[cit0028] Graneheim, U. H., & Lundman, B. (2004). Qualitative content analysis in nursing research: Concepts, procedures and measures to achieve trustworthiness. *Nurse Education Today*, 24(2), 105–112. 10.1016/j.nedt.2003.10.001 14769454

[cit0029] Harms, P. D., Credé, M., Tynan, M., Leon, M., & Jeung, W. (2017). Leadership and stress: A meta-analytic review. *The Leadership Quarterly*, 28(1), 178–194. 10.1016/j.leaqua.2016.10.006

[cit0030] Häusser, J. A., Mojzisch, A., Niesel, M., & Schulz-Hardt, S. (2010). Ten Years on: A review of recent research on the job demand–control (-support) model and psychological well-being. *Work & Stress*, 24(1), 1–35. 10.1080/02678371003683747

[cit0031] Hessels, J., Rietveld, C. A., Thurik, A. R., & Van der Zwan, P. (2018). Depression and entrepreneurial exit. *Academy of Management Perspectives*, 32(3), 323–339. 10.5465/amp.2016.0183

[cit0032] Hsieh, H.-F., & Shannon, S. E. (2005). Three approaches to qualitative content analysis. *Qualitative Health Research*, 15(9), 1277–1288. 10.1177/1049732305276687 16204405

[cit0033] Johnson, J. V., & Hall, E. M. (1988). Job strain, work place social support, and cardiovascular disease: A cross-sectional study of a random sample of the Swedish working population. *American Journal of Public Health*, 78(10), 1336–1342. 10.2105/AJPH.78.10.1336 3421392 PMC1349434

[cit0034] Joseph, D. L., Dhanani, L. Y., Shen, W., McHugh, B. C., & McCord, M. A. (2015). Is a happy leader a good leader? A meta-analytic investigation of leader trait affect and leadership. *The Leadership Quarterly*, 26(4), 557–576. 10.1016/j.leaqua.2015.04.001

[cit0035] Kaluza, A. J., Boer, D., Buengeler, C., & van Dick, R. (2020). Leadership behaviour and leader self-reported well-being: A review, integration and meta-analytic examination. *Work & Stress*, 34(1), 34–56. 10.1080/02678373.2019.1617369

[cit0036] Karasek, R., & Theorell, T. (1990). *Healthy Work: Stress, productivity, and the Reconstruction of working life*. Basic Books.

[cit0037] Kvale, S., & Brinkmann, S. (2009). *InterViews: Learning the craft of qualitative research interviewing*. SAGE.

[cit0038] Lechat, T., & Torrès, O. (2017). Stressors and satisfactors in entrepreneurial activity: An event-based, mixed methods study predicting small business owners’ health. *International Journal of Entrepreneurship & Small Business*, 32, 537–569. 10.1504/IJESB.2017.10007974

[cit0039] Lepine, J. A., Podsakoff, N. P., & Lepine, M. A. (2005). A meta-analytic test of the challenge stressor–hindrance stressor framework: An explanation for inconsistent relationships among stressors and performance. *Academy of Management Journal*, 48(5), 764–775. 10.5465/amj.2005.18803921

[cit0040] Lindgren, B.-M., Lundman, B., & Graneheim, U. H. (2020). Abstraction and interpretation during the qualitative content analysis process. *International Journal of Nursing Studies*, 108, 103632. 10.1016/j.ijnurstu.2020.103632 32505813

[cit0041] Lindholm, M. (2006). Working conditions, psychosocial resources and work stress in nurses and physicians in chief managers’ positions. *Journal of Nursing Management*, 14(4), 300–309. 10.1111/j.1365-2934.2006.00636.x 16629844

[cit0042] Li, W.-D., Schaubroeck, J. M., Xie, J. L., & Keller, A. C. (2018). Is being a leader a mixed blessing? A dual-pathway model linking leadership role occupancy to well-being. *Journal of Organizational Behavior*, 39(8), 971–989. 10.1002/job.2273

[cit0043] Lundqvist, D., Eriksson, A. F., & Ekberg, K. (2012). Exploring the relationship between managers’ leadership and their health. *Work*, 42(3), 419–427. 10.3233/WOR-2012-1395 22523020

[cit0044] Lundqvist, D., Eriksson, A. F., & Ekberg, K. (2018). Managers’ social support: Facilitators and hindrances for seeking support at work. *Work*, 59(3), 351–365. 10.3233/WOR-182690 29630581

[cit0045] Mäkiniemi, J.-P., Ahola, S., Nuutinen, S., Laitinen, J., & Oksanen, T. (2021). Factors associated with job burnout, job satisfaction and work engagement among entrepreneurs. A systematic qualitative review. *Journal of Small Business & Entrepreneurship*, 33(2), 219–247. 10.1080/08276331.2020.1764735

[cit0046] Mayer, C.-H., & Boness, C. (2011). Concepts of health and well-being in managers: An organizational study. *International Journal of Qualitative Studies on Health and Well-Being*, 6(4), 7143. 10.3402/qhw.v6i4.7143 PMC3200432 22028736

[cit0047] Mintzberg, H. (1973). *The nature of managerial work*. Harper & Row.

[cit0048] Morse, J. M. (2008). Confusing categories and themes. *Qualitative Health Research*, 18(6), 727–728. 10.1177/1049732308314930 18503013

[cit0049] Niedhammer, I., Bertrais, S., & Witt, K. (2021). Psychosocial work exposures and health outcomes: A meta-review of 72 literature reviews with meta-analysis. *Scandinavian Journal of Work, Environment & Health*, 47(7), 489–508. 10.5271/sjweh.3968 PMC8504166 34042163

[cit0050] Nielsen, K., Nielsen, M. B., Ogbonnaya, C., Känsälä, M., Saari, E., & Isaksson, K. (2017). Workplace resources to improve both employee well-being and performance: A systematic review and meta-analysis. *Work & Stress*, 31(2), 101–120. 10.1080/02678373.2017.1304463

[cit0051] Nixon, A. E., Mazzola, J. J., Bauer, J., Krueger, J. R., & Spector, P. E. (2011). Can work make you sick? A meta-analysis of the relationships between job stressors and physical symptoms. *Work & Stress*, 25(1), 1–22. 10.1080/02678373.2011.569175

[cit0052] Nyberg, A., Leineweber, C., & Magnusson Hanson, L. (2015). Gender differences in psychosocial work factors, work–personal life interface, and well-being among Swedish managers and non-managers. *International Archives of Occupational and Environmental Health*, 88(8), 1149–1164. 10.1007/s00420-015-1043-0 25761632

[cit0053] O’Gorman, C., Bourke, S., & Murray, J. A. (2005). The nature of managerial work in small growth-orientated businesses. *Small Business Economics*, 25(1), 1–16. 10.1007/s11187-005-4254-z

[cit0054] Omrane, A., Kammoun, A., & Seaman, C. (2018). Entrepreneurial burnout: Causes, consequences and way out. *FIIB Business Review*, 7(1), 28–42. 10.1177/2319714518767805

[cit0055] Owalla, B., Gherhes, C., Vorley, T., & Brooks, C. (2022). Mapping SME productivity research: A systematic review of empirical evidence and future research agenda. *Small Business Economics*, 58(3), 1285–1307. 10.1007/s11187-021-00450-3

[cit0056] Quick, J. C., Gavin, J. H., Cooper, C. L., Quick, J. D., & Gilbert, R. E. (2000). Executive health: Building strength, managing risks. *Academy of Management Executive*, 14(2), 34–46. 10.5465/ame.2000.3819304

[cit0057] Sanz-Vergel, E., Demerouti, A., & Sanz-Vergel, A. I. (2014). Burnout and work engagement: The JD–R approach. *Annual Review of Organizational Psychology and Organizational Behavior*, 1(1), 389–411. 10.1146/annurev-orgpsych-031413-091235

[cit0058] Sardeshmukh, S. R., Goldsby, M., & Smith, R. M. (2021). Are work stressors and emotional exhaustion driving exit intentions among business owners? *Journal of Small Business Management*, 59(4), 544–574. 10.1111/jsbm.12477

[cit0059] Siegrist, J. (1996). Adverse health effects of high-effort/low-reward conditions. *Journal of Occupational Health Psychology*, 1(1), 27–41. 10.1037/1076-8998.1.1.27 9547031

[cit0060] Siegrist, J. (2002). Effort-reward imbalance at work and health. In P. L. Perrewe & D. C. Ganster (Eds.), *Historical and Current perspectives on stress and health* (Vol. 2, pp. 261–291). Emerald Group Publishing Limited. 10.1016/S1479-3555(02)02007-3

[cit0061] Skagert, K., Dellve, L., & Ahlborg, G., Jr. (2012). A prospective study of managers’ turnover and health in a healthcare organization. *Journal of Nursing Management*, 20(7), 889–899. 10.1111/j.1365-2834.2011.01347.x 23050622

[cit0062] Skakon, J., Nielsen, K., Borg, V., & Guzman, J. (2010). Are leaders’ well-being, behaviours and style associated with the affective well-being of their employees? A systematic review of three decades of research. *Work & Stress*, 24(2), 107–139. 10.1080/02678373.2010.495262

[cit0063] Sonnentag, S. (2015). Dynamics of well-being. *Annual Review of Organizational Psychology and Organizational Behavior*, 2(1), 261–293. 10.1146/annurev-orgpsych-032414-111347

[cit0064] Stephan, U. (2018). Entrepreneurs’ mental health and well-being: A review and research agenda. *Academy of Management Perspectives*, 32(3), 290–322. 10.5465/amp.2017.0001

[cit0065] Tengblad, S. (2006). Is there a ‘new managerial work’? A comparison with Henry Mintzberg’s classic study 30 years later. *Journal of Management Studies*, 43(7), 1437–1461. 10.1111/j.1467-6486.2006.00651.x

[cit0066] Torrès, O., & Julien, P.-A. (2005). Specificity and denaturing of small business. *International Small Business Journal*, 23(4), 355–377. 10.1177/0266242605054049

[cit0067] Van der Doef, M., & Maes, S. (1999). The job demand-control (-support) model and psychological well-being: A review of 20 years of empirical research. *Work & Stress*, 13(2), 87–114. 10.1080/026783799296084

[cit0068] Visentin, D. C., Cleary, M., & Minutillo, S. (2020). Small business ownership and mental health. *Issues in Mental Health Nursing*, 41(5), 460–463. 10.1080/01612840.2020.1733871 32294389

[cit0069] Warr, P., & Nielsen, K. (2018). Wellbeing and work performance. In E. Diener, S. Oishi, & L. Tay (Eds.), *Handbook of well-being*. DEF Publishers. https://nobascholar.com

